# Randomised Controlled Crossover Trial Evaluating Two Caregiver‐Delivered Music Listening Interventions for Community‐Dwelling People with Dementia

**DOI:** 10.1002/alz.085541

**Published:** 2025-01-09

**Authors:** Lena M Hofbauer, Francisca S Rodriguez

**Affiliations:** ^1^ German Center for Neurodegenerative Diseases (DZNE), site Rostock/Greifswald, Greifswald Germany; ^2^ German Center for Neurodegenerative Diseases (DZNE), site Rostock/Greifswald, Greifswald, Mecklenburg‐Vorpommern Germany

## Abstract

**Background:**

Given that the majority of people with dementia live in their homes in the community and are primarily cared for by informal caregivers, there is a growing interest in developing interventions suitable for this setting. Further, it is important to establish how music selections can differentially affect PwD. In this pilot trial, we thus compare two caregiver‐delivered music listening MBIs with care‐as‐usual.

**Methods:**

17 PwD‐caregiver‐dyads were randomised to receive 6 weeks of ‘MBI A’ and ‘MBI B’, the order was crossed over. As control, half of the participants also underwent 6 weeks of care‐as‐usual. MBI A consisted of fast, positively valenced music while MBI B consisted of slow, positively valenced music. The music was provided on tablet or CD. Quantitative outcomes were cognition, well‐being, quality of life, and caregiver‐reported behavioural and psychiatric symptom severity and related distress. Outcomes were analysed in separate mixed‐effect models adjusted for baseline values, period, and intervention adherence. Dyads’ descriptions of experiences were narratively summarised.

**Results:**

Music listening was associated with an initial increase in quality of life. Further, MBI A was associated with superior delayed recall compared to MBI B (MBI A‐B: 1.04 [95% CI: 0.16, 1.92], *Hedge’s g_av_ = 0.70*). Dyads’ descriptions highlight differential positive effects of MBI A and B ‘in‐the‐moment’ of listening. Positive effects of MBI A included heightened mood and movement, MBI B was associated with relaxation. Dyads suggested that future iterations of the MBI may (i) be usable by PwD on their own, (ii) contain longer playlists, and (iii) be individualised.

**Conclusion:**

Overall, the music listening intervention created value for PwD, increasing quality of life and inducing positive experiences ‘in‐the‐moment’. The recall‐enhancing effect of listening to fast, positively valenced music over a prolonged period should be investigated further and may be relevant to targeted uses of music with PwD. While the MBI design used in the present investigation can be described as feasible overall, adapting it so that PwD may use the intervention independently may be a valuable next step.

